# Detection of Nucleocapsid Antibodies Associated with Primary SARS-CoV-2 Infection in Unvaccinated and Vaccinated Blood Donors

**DOI:** 10.3201/eid3008.240659

**Published:** 2024-08

**Authors:** Eduard Grebe, Mars Stone, Bryan R. Spencer, Akintunde Akinseye, David J. Wright, Clara Di Germanio, Roberta Bruhn, Karla G. Zurita, Paul Contestable, Valerie Green, Marion C. Lanteri, Paula Saa, Brad J. Biggerstaff, Melissa M. Coughlin, Steve Kleinman, Brian Custer, Jefferson M. Jones, Michael P. Busch

**Affiliations:** Vitalant Research Institute, San Francisco, California, USA (E. Grebe, M. Stone, C. Di Germanio, R. Bruhn, K.G. Zurita, B. Custer, M.P. Busch);; University of California, San Francisco (M. Stone, R. Bruhn, M.C. Lanteri, B. Custer, M.P. Busch);; American Red Cross, Rockville, Maryland, USA (B.R. Spencer, P. Saa);; Westat, Rockville (A. Akinseye, D. Wright); QuidelOrtho, Rochester, New York, USA (P. Contestable);; Creative Testing Solutions, Tempe, Arizona, USA (V. Green, M.C. Lanteri);; Centers for Disease Control and Prevention, Fort Collins, Colorado, USA (B.J. Biggerstaff);; Centers for Disease Control and Prevention, Atlanta, Georgia, USA (M.M. Coughlin, J.M. Jones);; University of British Columbia, Vancouver, British Columbia, Canada (S. Kleinman)

**Keywords:** COVID-19, SARS-CoV-2, viruses, respiratory infections, zoonoses, vaccine-preventable diseases, blood safety, United States

## Abstract

Nucleocapsid antibody assays can be used to estimate SARS-CoV-2 infection prevalence in regions implementing spike-based COVID-19 vaccines. However, poor sensitivity of nucleocapsid antibody assays in detecting infection after vaccination has been reported. We derived a lower cutoff for identifying previous infections in a large blood donor cohort (N = 142,599) by using the Ortho VITROS Anti-SARS-CoV-2 Total-N Antibody assay, improving sensitivity while maintaining specificity >98%. We validated sensitivity in samples donated after self-reported swab-confirmed infection diagnoses. Sensitivity for first infections in unvaccinated donors was 98.1% (95% CI 98.0–98.2) and for infection after vaccination was 95.6% (95% CI 95.6–95.7) based on the standard cutoff. Regression analysis showed sensitivity was reduced in the Delta compared with Omicron period, in older donors, in asymptomatic infections, <30 days after infection, and for infection after vaccination. The standard Ortho N antibody threshold demonstrated good sensitivity, which was modestly improved with the revised cutoff.

In the United States, as in many countries, convenience sample serosurveillance studies (e.g., in blood donors) have demonstrated that most of the population has SARS-CoV-2 antibodies from vaccination, infection, or both (*1*–*3*). Therefore, continued surveillance using serologic tests requires robust detection of first infections in vaccinated persons (infection after vaccination) and reinfections to yield meaningful estimates of infection incidence. Serologic detection of nucleocapsid antibodies has been a critical tool to detect previous SARS-CoV-2 infection and discriminate between vaccine- and infection-induced antibody reactivity in the context of spike-based vaccines.

The National Blood Donor Cohort (NBDC), a longitudinal study sponsored by the Centers for Disease Control and Prevention (CDC), was conducted in partnership with the 2 largest US blood collectors (Vitalant and the American Red Cross) and their central testing laboratory Creative Testing Solutions (M. Stone et al., unpub. data). We classified participating donors into 4 groups on the basis of infection and vaccination status as of mid-2021: not previously infected or vaccinated, previously infected, previously vaccinated, or both previously infected and vaccinated. An earlier iteration of this program (the National Blood Donor Serosurvey) conducted serial monthly cross-sectional serosurveys during July 2020–December 2021 (*4*–*6*) to provide population-weighted seroprevalence estimates. However, because vaccination rates increased in 2021, the percentage of donations with vaccine-induced, infection-induced, or both vaccine-induced and infection-induced spike antibody reactivity approached 95%, and rates of infection-induced N antibody reactivity exceeded 75% in the United States by the end of 2022 (*1*,*7*), thus diminishing the value of cross-sectional serosurveillance. Important objectives of the NBDC included continued monitoring of SARS-CoV-2 infection incidence and vaccine- and infection-induced seroprevalence in the context of endemic SARS-CoV-2 transmission and increasing frequency of infection after vaccination and reinfections (M. Stone et al., unpub. data).

Several studies have suggested that the sensitivity of nucleocapsid antibody serologic tests for detecting previous SARS-CoV-2 infection is reduced in vaccinated persons compared with unvaccinated persons (*8*–*10*; H.J. Whitaker et al., unpub. data, https://doi.org/10.1101/2021.10.25.21264964). Significantly reduced nucleocapsid antibody reactivity has been reported in previously vaccinated persons with PCR-confirmed infections compared with infections in unvaccinated persons (H.J. Whitaker et al., unpub. data). Moderna mRNA vaccine trial data showed lower rates of nucleocapsid antibody seropositivity after PCR-confirmed infection among vaccine recipients compared with placebo recipients (40.4% vs. 93.4%) (*8*). A blunted nucleocapsid antibody response for infection after vaccination, and consequently reduced sensitivity of nucleocapsid antibody serology, may result from suppression of viral replication attributable to existing spike antibodies and an associated anamnestic response (*11*). In addition, studies relying on nucleocapsid IgG detection (*12*) may be confounded by rapidly waning antibodies below the limit of detection (i.e., seroreversion) (*13*–*16*).

We previously demonstrated good performance of the Ortho VITROS Anti-SARS-CoV-2 Total N Antibody (Ortho nucleocapsid antibody) assay (QuidelOrtho, https://www.quidelortho.com) and Roche Elecsys NC Anti-SARS-CoV-2 (Roche nucleocapsid antibody) assay (Roche, https://www.roche.com) for serosurveillance applications, without differentiating infections in vaccinated and unvaccinated persons (*17*). In this study, to increase sensitivity while maintaining high specificity for serologic detection of infection after vaccination, we derived a revised reactive versus nonreactive cutoff for those assays. In addition, for the Ortho nucleocapsid antibody assay we sought to validate the sensitivity of both the manufacturer’s recommended and our revised cutoff for identifying first infections in vaccinated and unvaccinated donors who self-reported swab-confirmed infections. We then assessed factors influencing detection of nucleocapsid antibodies and assessed the durability of antibody detection after primary infection.

## Materials and Methods

### Study Population

We identified repeat blood donors from 2 national blood collection organizations (Vitalant and American Red Cross) who had known prior SARS-CoV-2 infection and COVID-19 vaccination status determined during June 2020–July 2021, when all donations were tested for SARS-CoV-2 antibodies and donors reported vaccination status at the time of donation. Eligible donors were those presenting >2× during the screening period and meeting all blood donor eligibility criteria. The NBDC includes 142,599 repeat blood donors. We based eligibility screening on donations tested with Ortho VITROS Anti-SARS-CoV-2 (spike) antibody assay and Roche nucleocapsid antibody assay, and we retained all spike antibody–reactive samples (*5*,*6*,*18*,*19*). During follow-up from July 2021 through December 2022, we identified donation specimens in real time and stored them frozen at –20°C. In 2022, we tested 1 donation specimen per donor per quarter by using the Ortho VITROS Anti-SARS-CoV-2 IgG Quantitative Test and the Ortho nucleocapsid antibody assay at Creative Testing Solutions and Vitalant Research Institute. We captured self-reported vaccination status at each donation as part of routine donation procedures. We invited all cohort donors to respond to electronic surveys on vaccination history, infection history, and clinical outcomes of infections; the overall response rate was 46.5%. NBDC seroprevalence estimates have been published (*1*).

### Analysis and Statistical Methods

We conducted all analyses by using the SAS System version 9.4 (SAS Institute, https://www.sas.com). In total, we conducted 6 specific statistical analyses.

#### Derivation of Revised Nonreactive versus Reactive Cutoff for Ortho Nucleocapsid Antibody Assay

To detect infections serologically with optimal sensitivity, we derived a revised cutoff by using receiver operating characteristic (ROC) curve analysis (Appendix). We consequently defined gray zone reactivity as reactivity above the revised threshold and below the standard threshold (0.395<signal-to-cutoff ratio [S/CO]<1.0).

#### Effect of Vaccination Status on Nucleocapsid Antibody Reactivity

To assess whether first infections after vaccination were associated with reduced postinfection nucleocapsid antibody reactivity compared with first infections in unvaccinated donors, we evaluated Ortho nucleocapsid antibody assay reactivity distributions after infection in 2 groups: serologically identified putative first infections, defined as the first donation sample for each donor in which nucleocapsid antibody reactivity was above the revised cutoff, among all donors in the NBDC; and first survey-reported swab-confirmed infections. For the first group, we based vaccination status on self-report at the time of donation. For the second group, we defined swab-confirmed infections as infections confirmed by viral antigen or PCR testing or by physician diagnosis (presumed positive swab-based test) and vaccination status at the time of infection on survey-reported vaccinations. For both groups, we stratified donation samples by vaccination status (vaccinated vs. unvaccinated) at the time of infection and by period (the Delta variant era, July–December 2021, vs. the Omicron era, January–December 2022 [*20*]) and stratified them further by quarter. We calculated the proportions of donation samples with reactivity in the gray zone for each group.

#### Sensitivity of Manufacturer’s Recommended and Revised Cutoffs for Detection of First Infections

To validate sensitivity, we identified survey-reported swab-confirmed first SARS-CoV-2 infections. We classified an infection as occurring in an unvaccinated donor if the donor had not reported any vaccination before the date of diagnosed infection, and we defined an infection as infection after vaccination if it occurred >14 days after completion of an approved primary vaccination series (1 dose of the J&J/Janssen vaccine [https://www.jnj.com] or 2 doses of either the Pfizer-BioNtech [https://www.pfizer.com] or Moderna [https://www.modernatx.com] mRNA vaccines). For cases to be included in this analysis, >1 donation sample had to have been collected 14–180 days after diagnosis with no prior nucleocapsid antibody reactivity above the standard cutoff. We identified a total of 2,751 swab-confirmed first infections in unvaccinated donors and 8,187 swab-confirmed first infections that were infection after vaccination. For a secondary, more restrictive analysis, we only included infection after vaccination if a postvaccination spike antibody–reactive, nucleocapsid antibody–nonreactive sample had been collected before infection, demonstrating vaccine-induced spike antibodies seroconversion in the absence of infection-induced nucleocapsid antibodies. We excluded infections occurring after only 1 mRNA vaccination dose or <14 days after completion of a vaccination series. We identified 5,079 infection after vaccination cases for this analysis. We included only 1 postinfection sample per case in either analysis.

We estimated the sensitivity of both the manufacturer’s recommended and our revised cutoffs on the Ortho nucleocapsid antibody assay in first donation samples after swab-confirmed infections, stratified by vaccination status of the donor at the time of infection. In addition, we stratified infections according to the variant era (Delta period vs. Omicron period), donor age (<65 years vs. *>*65 years), and whether the infection was associated with >1 self-reported symptom. We defined sensitivity as the proportion of samples that were reactive and calculated 95% CIs by using the Wilson score method. We assessed differences in sensitivity for different strata by using the binomial exact test. We assessed differences in sensitivity associated with different cutoffs computed on the same stratum by computing a p value for the difference in the Youden’s J statistic associated with each cutoff.

#### Factors Associated with Nucleocapsid Antibody Seroconversion after Swab-Confirmed Infection

We performed bivariate and multivariable logistic regression to assess the effect of vaccination status, timing of sample collection relative to infection, donor demographics (age and sex), presence of symptoms, and variant era on nucleocapsid antibody detection. We included samples collected <14 days or >180 days after swab-confirmed infection because the model adjusted for time from infection to sample collection. We computed unadjusted odds ratios (ORs) and adjusted odds ratios (aORs) from logistic regression. After assessing ORs, we combined the vaccination status and timing variables for the multivariable regression.

#### Durability of Nucleocapsid Antibody Detection

We assessed durability of nucleocapsid antibody detection after primary infection in unvaccinated and vaccinated donors by examining the proportion of primary infections detectable by time from swab-confirmed infection to sample collection (0–13, 14–30, 31–60, 61–90, 91–180, 181–365, and >365 days). To account for multiple observations per time bin per donor, we weighted observations so that donors were equally weighted within each time bin, regardless of the number of observations. We computed 95% CIs by using the Wilson score method.

#### Effect of Adjustment for Nucleocapsid Antibody Sensitivity on Seroprevalence Estimates

To assess the potential effect of imperfect sensitivity and specificity on estimates of infection rates among vaccinated persons, we compared adjusted and unadjusted estimates of the proportion of vaccinated donors (not previously infected) who experienced infection after vaccination during 3 periods in the NBDC: quarter (Q) 2 2021–Q1 2022, Q1–Q2 2022, and Q2–Q3 2022. We cannot estimate the proportion of infections that were asymptomatic from survey data because diagnostic testing is largely driven by the presence of symptoms; therefore, for the purposes of this model, we used an estimated proportion of infections after vaccination that are asymptomatic of 32.4% (*21*). We then adjusted for a weighted average of symptomatic- and asymptomatic-specific sensitivity estimates and for specificity estimated using prepandemic samples. We computed the adjusted proportion of persons infected during each period by using the estimator derived by Rogan and Gladen (*22*), and we based 95% CIs on parametric bootstrapping (10,000 iterations) of the proportion of tests that were reactive, sensitivity, and specificity (treated as binomially distributed and incorporating the uncertainty arising from limited sample size).

### Ethics Considerations

All blood donors consented to use of deidentified, residual specimens for further research purposes. Consistent with the policies and guidance of the University of California–San Francisco Institutional Review Board, Vitalant Research Institute self-certified the use of deidentified donations in this study as not meeting the criteria for human subjects research. CDC investigators reviewed and relied on this determination as consistent with applicable federal law and CDC policy. The donor surveys conducted by Vitalant Research Institute and American Red Cross were conducted under protocols supervised and approved by the Advarra and American Red Cross institutional review boards, respectively, and linked to biospecimens in deidentified form.

## Results

### Revised Cutoff for Detecting Previous Infection with Ortho Nucleocapsid Antibody Assay

The nonreactive versus reactive threshold on the Roche nucleocapsid antibody cutoff index that maximized Youden’s J statistic was >0.205, and this optimized cutoff was used in defining cases for the Ortho ROC curve analysis. The ROC-optimized threshold on the Ortho nucleocapsid antibody assay was S/CO >0.395, which had a sensitivity of 98.7% and a specificity of 98.7% in the Ortho optimization sample set. The area under the ROC curve was 0.994 (Appendix).

### Effect of Vaccination Status on Nucleocapsid Antibody Reactivity

**Figure 1:**
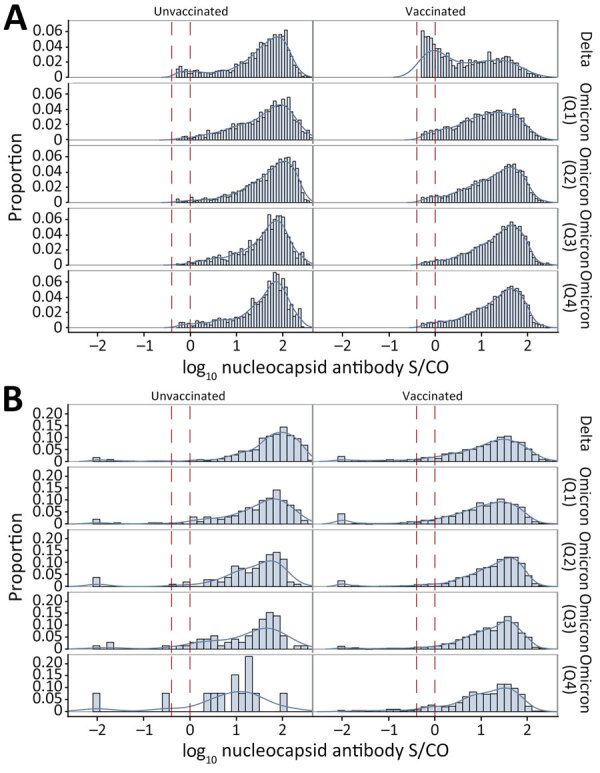
Nucleocapsid antibody signal intensity distributions observed in vaccinated and unvaccinated blood donors after primary SARS-CoV-2 infection, United States, July 2021–December 2022. A) Reactivity of putative serologically identified infections at the first longitudinal sample showing reactivity above the reduced cutoff (gray zone reactivity, S/CO ratio>0.395<1), by vaccination status and variant era (6,555 unvaccinated donors [left] and 22,217 vaccinated donors [right]). B) Reactivity at the first sample collected after self-reported swab-confirmed infection (14–180 days postinfection), by vaccination status and variant era (2,751 unvaccinated donors [left] and 8,187 vaccinated donors [right]). Vertical dashed lines indicate the gray zone of nucleocapsid antibodies. Q1, January–March 2022; Q2, April–June 2022; Q3, July–September 2022; Q4, October–December 2022. S/CO, signal-to-cutoff ratio.

We calculated distributions of Ortho nucleocapsid antibody assay S/COs in first longitudinal samples with reactivity above the revised cutoff (S/CO ≥0.395 [i.e., putative first infections]) from previously uninfected donors (based on negative Ortho nucleocapsid antibody results [S/CO <0.395] in all previous longitudinal samples), by vaccination status and variant era (Figure 1). 

During the Delta era, 35.2% of serologically identified infections after vaccination showed gray zone reactivity (0.395<S/CO< 10) compared with 7.5% of serologically identified primary infections in unvaccinated donors, declining to 3.8% for infections after vaccination and 2.7% for primary infections in unvaccinated donors by Q4 2022 (Omicron period Q4) (Figure 1, panel A). Among survey respondents with swab-confirmation infection, we did not observe a similar increased proportion of gray zone nucleocapsid antibody reactivity in the Delta period; most had reactivity above the standard cutoff (Figure 1, panel B). The donation specimens (Figure 1, panel B) correspond to the specimens used in assessing sensitivity for the 2 cutoffs.

### Sensitivity for Detection of Swab-Confirmed Primary Infections

Overall sensitivity of the Ortho nucleocapsid antibody assay manufacturer’s cutoff was 98.1% (95% CI 98.0%–98.2%) for detection of first infections in unvaccinated donors and 95.6% (95% CI 95.6%–95.7%) for detection of first infections after vaccination (Table). Sensitivity was increased when using the revised cutoff, to 98.4% (95% CI 98.4%–98.5%) in unvaccinated donors and to 97.0% (95% CI 96.9%–97.0%) for infections after vaccination. Although sensitivity is necessarily increased by reducing the cutoff from the manufacturer’s suggestion, Youden’s J index is not statistically improved by the reduced cutoff (p = 0.13 based on a 1-tailed test). Sensitivity for detection of infection after vaccination using the standard cutoff was higher during the Omicron era (96.0% [95% CI 95.9%–96.0%) than the Delta era (93.9% [95% CI 93.5%–95.4%]; p = 0.001) and was higher for detecting symptomatic than asymptomatic infections after vaccination (96.2% [95% CI 96.1%–96.2%] vs. 90.1% [95% CI 88.5%–91.7%]; p<0.0001). A secondary sensitivity analysis using a more restrictive case definition of infection after vaccination, which required spike antibody seroconversion after vaccination, showed similar patterns (Appendix Table 1).

**Table:** Sensitivity of SARS-CoV-2 nucleocapsid antibody assay for detection of first infections in unvaccinated and vaccinated donors, United States, July 2021–December 2022*

Characteristic	Sensitivity for detection of first infections in unvaccinated donors				Sensitivity for detection of first infections in vaccinated donors		
	No. donors	Manufacturer’s cutoff,† % (95% CI)	Revised cutoff,‡ % (95% CI)		No. donors	Manufacturer’s cutoff,† % (95% CI)	Revised cutoff,‡ % (95% CI)
Overall	2,751	98.1 (98.0–98.2)	98.4 (98.4–98.5)		8,187	95.6 (95.6–95.7)	97.0 (96.9–97.0)
Delta: Jul–Dec 2021	1,343	98.4 (98.2–98.5)	98.5 (98.4–98.6)		1,349	93.9 (93.5–94.4)	95.3 (94.9–95.6)
Omicron: Jan–Dec 2022	1,408	97.9 (97.7–98.0)	98.4 (98.3–98.5)		6,838	96.0 (95.9–96.0)	97.3 (97.3–97.4)
Age <65 y	2,225	98.2 (98.1–98.2)	98.4 (98.4–98.5)		5,194	96.2 (96.1–96.3)	97.3 (97.2–97.3)
Age >65 y	526	97.9 (97.5–98.3)	98.5 (98.2–98.8)		2,993	94.7 (94.5–94.9)	96.5 (96.4–96.6)
Symptomatic§	2,430	98.4 (98.3–98.4)	98.7 (98.7–98.8)		7,416	96.2 (96.1–96.2)	97.5 (97.5–97.6)
Asymptomatic§	208	96.6 (95.0–98.3)	96.6 (95.0–98.3)		627	90.1 (88.5–91.7)	91.2 (89.8–92.6)

*Proportion reactive in the first sample collected after reported swab-confirmed infection, collected 14 to 180 days postinfection. †Signal-to-cutoff ratio >1.000. ‡Signal-to-cutoff ratio >0.395. §Symptomatic or asymptomatic status could not be ascertained for all infections because of incomplete survey responses.

### Factors Associated with Nucleocapsid Antibody Seroconversion after Swab-Confirmed Infection

**Figure 2:**
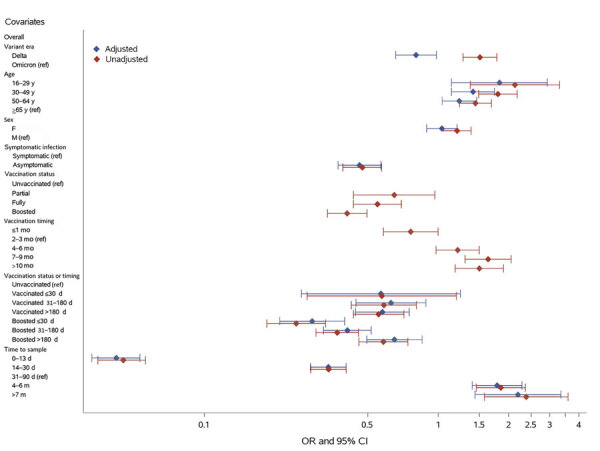
Factors influencing nucleocapsid antibody seroconversion after swab-confirmed first SARS-CoV-2 infections among vaccinated and unvaccinated blood donors, United States, July 2021–December 2022. ORs and 95% CIs from logistic regression are shown. In the multivariable regression model (adjusted ORs), the categories for certain variables have been grouped together; the vaccination status at the time of infection and timing of most recent vaccine before infection were combined in the vaccination status or timing variable, and in the variable for time from infection to tested sample, the groups for samples collected 7–12 months and >1 year postinfection were combined. Number of samples in each group, the proportion of nucleocapsid antibody–reactive samples, and ORs are shown in Appendix Table 2. OR, odds ratio; ref, referent.

On first samples collected after first swab-confirmed infection, bivariate logistic regression showed that infection during the Delta era, age <65 years, female sex, symptomatic infection, being unvaccinated at the time of infection, longer time between vaccination and infection, and longer intervals between infection and sample collection were all statistically significantly associated with increased probability of detection (Figure 2; Appendix Table 2). Of note, the percentage detected in all time to sample categories, other than donation samples collected <14 days postinfection (dpi) (48.9%), ranged from 87.7% to 98.1%.

In multivariable logistic regression, infection during the Delta period (vs. Omicron period) remained statistically significant, but the direction of effect changed to reduced detection (aOR 0.80 [95% CI 0.66–0.98]) from increased detection in bivariate analysis (OR 1.51 [95% CI 1.28–1.78]), possibly because variant era (calendar time) is also strongly associated with vaccination status. Younger age groups had higher odds of detection than donors >65 years of age, whereas donor sex was not significantly associated with detection in the multivariable analysis. Asymptomatic infection was significantly associated with reduced detection (aOR 0.46, 95% CI 0.37–0.57). Being vaccinated at the time of infection significantly reduced detection compared with being unvaccinated (aORs <1), with the exception of primary vaccination <30 days before infection, which was not statistically significant. Vaccination reduced detection compared with no vaccination, and more recent receipt of either a primary vaccination series (31–180 days before infection) or a booster vaccination (<30 days or 31–180 days before infection) was associated with reduced odds of detection than when infections occurred >180 days since the most recent vaccine. Compared with sample collection 31–90 dpi, sample collection <14 days (aOR 0.04 [95% CI 0.03–0.05]) and 14–30 days (aOR 0.34 [95% CI 0.28–0.40]) after infection were associated with greatly reduced detection, whereas sample collection 3–6 months (aOR 1.79 [95% CI 1.40–2.28]) or >7 months (aOR 2.19 [95% CI 1.44–3.35]) after infection were associated with increased detection (Figure 2; Appendix Table 2).

### Durability of Nucleocapsid Antibody Detection

**Figure 3:**
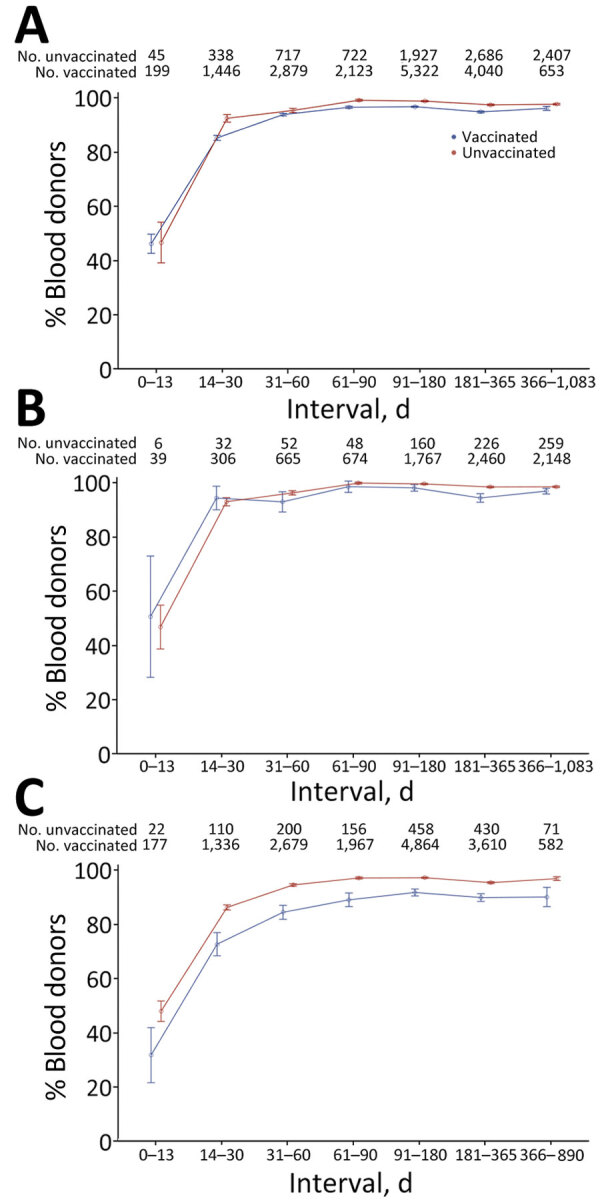
Sensitivity of nucleocapsid antibody serologic tests by time from swab-confirmed infection to sample collection in vaccinated and unvaccinated blood donors, using the manufacturer’s recommended cutoff, United States, July 2021–December 2022. The percentage of donors showing reactivity in first or subsequent samples after swab-confirmed infection is shown. A) Reactive proportions stratified by vaccination status at the time of infection. B) Reactive proportions for infections in unvaccinated donors stratified by reported symptoms. C) Reactive proportions for infections in vaccinated donors, stratified by reported symptoms. To account for multiple observations per time bin, observations were weighted so that donors were equally weighted within each time bin, regardless of the number of observations. Error bars indicate median and maximum durations of follow-up for each group.

Nucleocapsid antibody reactivity was detected in less than half of specimens collected <14 dpi, >80% of specimens collected 14–30 dpi, and >90% of specimens collected >90 dpi in donors who were vaccinated and unvaccinated at the time of infection. We observed no decline in percentage detected in later time bins, including >1 year postinfection. The proportion detected was slightly lower in vaccinated donors and for asymptomatic infections at all times after infection (Figure 3).

### Effect of Adjustment for Nucleocapsid Antibody Sensitivity on Seroprevalence Estimates

Estimates of the proportion of vaccinated donors who had become infected in each time period (using the nucleocapsid antibody test), adjusted for sensitivity and specificity, and assuming that 32.4% of infections were asymptomatic, differed little from unadjusted estimates. Adjusted estimated infection rates increased in each period by 0.2–0.9 percentage points, or proportionally by 1.5%–4.0% (Appendix Table 3).

## Discussion

Despite reports of sensitivity as low as 40% for serologic detection of infection after vaccination (*8*), our findings demonstrate sensitivity for detection of swab-confirmed first infections >98% among unvaccinated persons and >95% among vaccinated persons and supports use of the manufacturer’s recommended cutoff for identifying previous infections in vaccinated and unvaccinated persons. Timing of sample collection after infection affected sensitivity (we observed poorer sensitivity <30 dpi); thus, timing of sample collection must be considered when interpreting previous reports.

In our validation of sensitivity for detection of first infections, the number of infections in vaccinated donors greatly exceeded those in unvaccinated donors in the study period, a function of high donor vaccination rates. This finding demonstrates the importance of sensitive detection of infection after vaccination in SARS-CoV-2 serosurveillance programs.

The revised cutoff for the Ortho nucleocapsid antibody assay offered minimal improvement in sensitivity to detect infection after vaccination compared with the manufacturer’s cutoff. Our ROC analysis equally weighted sensitivity and specificity, an approach appropriate for surveillance applications, but potentially less appropriate for clinical applications prioritizing specificity. The revised cutoff was not associated with a statistical improvement in Youden’s J statistic and only minimally increased sensitivity. The effect of adjusting seroprevalence estimates in vaccinated NBDC donors for sensitivity and specificity was modest (Appendix Table 3), not exceeding a proportional effect of 4% on point estimates. This finding indicates that the standard assay cutoff for infection after vaccination detection performed sufficiently.

Recent receipt of primary or booster vaccinations reduced the likelihood of nucleocapsid antibody seroconversion after infection. Multivariable regression showed that recent receipt of an additional vaccine (booster) dose was associated with reduced detection, but the timing of primary vaccination relative to infection affected detection less. A study in Japan showed similar results, indicating reduced sensitivity to detect infection within 1–2 months of a third mRNA COVID-19 vaccine dose (sensitivity 78%) but high sensitivity for infections occurring >3 months after the second or >4 months after the third dose (*23*). The Moderna vaccine trial data (*8*) were selected for infections after vaccination occurring soon after vaccination and were collected relatively soon after infection, likely contributing to poor sensitivity in that study (*8*). Our data confirmed relatively poor sensitivity in specimens collected within 1 month of infection.

Because much of the lack of detection observed in our study occurred at times soon after infection, a longitudinal cohort study probably would detect many of the infections missed at the first postinfection specimen at later timepoints using specimens from those persons. Despite some waning of nucleocapsid antibody levels after infection, longer-term durability of antibody detection >1 year after infection confirmed earlier findings by our group of robust durability of detection using nucleocapsid direct antigen sandwich assays (*13*). We observed substantially reduced detection in the first month after infection, especially during the first 14 days, when <50% of recently infected persons demonstrated nucleocapsid antibody seroconversion but observed very good detection at later timepoints. Nucleocapsid IgG assays used in numerous serosurveillance studies (*14*, *24*–*28*) show more rapid waning in antibody signal than nucleocapsid total Ig assays (*13*) and thus require adjustments for seroreversion in estimating cumulative incidence (*14*). So-called direct immunoassays (i.e., antigen sandwich format total Ig assays), are more sensitive to increasing antibody affinity than IgG assays, which probably explains the more durable reactivity associated with antibody maturation and persistence postinfection despite waning in IgG concentrations (*29*). Although rapidly waning IgG assays may be less appropriate for serosurveillance aimed at documenting cumulative incidence than total Ig assays, they may have advantages for detecting reinfections based on antibody boosting and as correlates of protection (*30*).

A limitation of this study was that the case definition of infection in the validation data was based on self-reported diagnosed infection, without active surveillance of the cohort for asymptomatic infection. As a result, most of survey-reported swab-confirmed infections in the validation set were associated with COVID-19 symptoms (92%). In contrast, a meta-analysis of Omicron infections estimated that 32.4% of infections were asymptomatic (*31*). This limitation may result in a slight upward bias in our overall sensitivity estimates. However, we found that adjusting for sensitivity to detect symptomatic and asymptomatic infection after vaccination had a modest effect on seroprevalence estimates. A further limitation is that blood donors are not fully representative of the general population; they generally are healthier and more likely to be vaccinated and to receive additional doses (*1*,*32*). Furthermore, vaccination and infection history were self-reported in donor surveys and not confirmed by healthcare records; only 46.5% of cohort participants responded to surveys and could be included in this study, which may have resulted in a biased sample.

Our study demonstrates that detection of first SARS-CoV-2 infections using the Ortho nucleocapsid total Ig antibody assay was robust in vaccinated and unvaccinated donors, indicating overall sensitivities >95%. We also found good durability of nucleocapsid antibody detection for up to >1 year after infection. Seroprevalence studies using this assay can accurately estimate the proportion of persons who have been infected with SARS-CoV-2 >1 times. Several factors affect the likelihood of nucleocapsid antibody seroconversion after first infection, including receipt of primary and additional vaccinations, sampling shortly after infection, and asymptomatic infection, although the effect of these factors was relatively small. Revising the cutoff improved sensitivity only modestly; therefore, use of the manufacturer’s recommended cutoff is likely appropriate for most serosurveillance studies.

Appendix Additional information about detection of nucleocapsid antibodies associated with primary SARS-CoV-2 infection in unvaccinated and vaccinated blood donors.

## References

[1] Jones JM, Manrique IM, Stone MS, Grebe E, Saa P, Germanio CD, et al. Estimates of SARS-CoV-2 seroprevalence and incidence of primary SARS-CoV-2 infections among blood donors, by COVID-19 vaccination status—United States, April 2021–September 2022. MMWR Morb Mortal Wkly Rep. 2023;72:601–5. 10.15585/mmwr.mm7222a3 37262007 PMC10243484

[2] Busch MP, Stone M. Serosurveillance for severe acute respiratory syndrome coronavirus 2 (SARS-CoV-2) incidence using global blood donor populations. Clin Infect Dis. 2021;72:254–6. 10.1093/cid/ciaa1116 33501953 PMC7454349

[3] O’Brien SF, Lieshout-Krikke RW, Lewin A, Erikstrup C, Steele WR, Uzicanin S, et al.; Surveillance, Risk Assessment, Policy Sub-group of the ISBT Transfusion Transmitted Infectious Diseases Working Party. Research initiatives of blood services worldwide in response to the covid-19 pandemic. Vox Sang. 2021;116:296–304. 10.1111/vox.12995 33165917

[4] Stone M, Di Germanio C, Wright DJ, Sulaeman H, Dave H, Fink RV, et al.; NHLBI Recipient Epidemiology and Donor Evaluation Study-IV-Pediatric (REDS-IV-P). Use of US blood donors for national serosurveillance of severe acute respiratory syndrome coronavirus 2 antibodies: basis for an expanded national donor serosurveillance program. Clin Infect Dis. 2022;74:871–81. 10.1093/cid/ciab537 34111244 PMC8406874

[5] Jones JM, Stone M, Sulaeman H, Fink RV, Dave H, Levy ME, et al. Estimated US infection- and vaccine-induced SARS-CoV-2 seroprevalence based on blood donations, July 2020–May 2021. JAMA. 2021;326:1400–9. 10.1001/jama.2021.15161 34473201 PMC8414359

[6] Jones JM, Opsomer JD, Stone M, Benoit T, Ferg RA, Stramer SL, et al. Updated US infection- and vaccine-induced SARS-CoV-2 seroprevalence estimates based on blood donations, July 2020–December 2021. JAMA. 2022;328:298–301. 10.1001/jama.2022.9745 35696249 PMC9194752

[7] Centers for Disease Control and Prevention. 2022 nationwide COVID-19 infection- and vaccination-induced antibody seroprevalence (blood donations). 2023 [cited 2023 Dec 23]. https://covid.cdc.gov/covid-data-tracker/#nationwide-blood-donor-seroprevalence-2022

[8] Follmann D, Janes HE, Buhule OD, Zhou H, Girard B, Marks K, et al. Antinucleocapsid antibodies after SARS-CoV-2 infection in the blinded phase of the randomized, placebo-controlled mRNA-1273 COVID-19 vaccine efficacy clinical trial. Ann Intern Med. 2022;175:1258–65. 10.7326/M22-1300 35785530 PMC9258784

[9] Dhakal S, Yu T, Yin A, Pisanic N, Demko ZO, Antar AAR, et al. Reconsideration of anti-nucleocapsid IgG antibody as a marker of SARS-CoV-2 infection post-vaccination for mild COVID-19 patients. Open Forum Infect Dis. 2022;10:c677. 10.1093/ofid/ofac677 36655185 PMC9835753

[10] Dalai SC, Dines JN, Snyder TM, Gittelman RM, Eerkes T, Vaney P, et al. Clinical validation of a novel T-cell receptor sequencing assay for identification of recent or prior severe acute respiratory syndrome coronavirus 2 infection. Clin Infect Dis. 2022;75:2079–87. 10.1093/cid/ciac353 35521791 PMC9129217

[11] Zuo J, Dowell AC, Pearce H, Verma K, Long HM, Begum J, et al. Robust SARS-CoV-2-specific T cell immunity is maintained at 6 months following primary infection. Nat Immunol. 2021;22:620–6. 10.1038/s41590-021-00902-8 33674800 PMC7610739

[12] Anderson M, Stec M, Gosha A, Mohammad T, Boler M, Tojo Suarez R, et al. Longitudinal severe acute respiratory syndrome coronavirus 2 vaccine antibody responses and identification of vaccine breakthrough infections among healthcare workers using nucleocapsid immunoglobulin G. J Infect Dis. 2022;226:1934–42. 10.1093/infdis/jiac420 36263799 PMC9619786

[13] Stone M, Grebe E, Sulaeman H, Di Germanio C, Dave H, Kelly K, et al. Evaluation of commercially available high-throughput SARS-CoV-2 serologic assays for serosurveillance and related applications. Emerg Infect Dis. 2022;28:672–83. 10.3201/eid2803.211885 35202525 PMC8888213

[14] Buss LF, Prete CA Jr, Abrahim CMM, Mendrone A Jr, Salomon T, de Almeida-Neto C, et al. Three-quarters attack rate of SARS-CoV-2 in the Brazilian Amazon during a largely unmitigated epidemic. Science. 2021;371:288–92. 10.1126/science.abe9728 33293339 PMC7857406

[15] Erikstrup C, Laksafoss AD, Gladov J, Kaspersen KA, Mikkelsen S, Hindhede L, et al. Seroprevalence and infection fatality rate of the SARS-CoV-2 Omicron variant in Denmark: A nationwide serosurveillance study. Lancet Reg Health Eur. 2022;21:100479. 10.1016/j.lanepe.2022.100479 35959415 PMC9355516

[16] Hønge BL, Hindhede L, Kaspersen KA, Harritshøj LH, Mikkelsen S, Holm DK, et al. Long-term detection of SARS-CoV-2 antibodies after infection and risk of re-infection. Int J Infect Dis. 2022;116:289–92. 10.1016/j.ijid.2022.01.041 35077881 PMC8783526

[17] Sulaeman H, Grebe E, Dave H, McCann L, Di Germanio C, Sanghavi A, et al. Evaluation of Ortho VITROS and Roche Elecsys S and NC Immunoassays for SARS-CoV-2 Serosurveillance Applications. Microbiol Spectr. 2023;11:e0323422. 10.1128/spectrum.03234-22 37347180 PMC10434072

[18] Fink RV, Fisher L, Sulaeman H, Dave H, Levy ME, McCann L, et al. How do we…form and coordinate a national serosurvey of SARS-CoV-2 within the blood collection industry? Transfusion. 2022;62:1321–33. 10.1111/trf.16943 35607854 PMC9348230

[19] Busch MP, Stramer SL, Stone M, Yu EA, Grebe E, Notari E, et al. Population-weighted seroprevalence from severe acute respiratory syndrome coronavirus 2 (SARS-CoV-2) infection, vaccination, and hybrid immunity among US blood donations from January to December 2021. Clin Infect Dis. 2022;75(Suppl 2):S254–63. 10.1093/cid/ciac470 35684973 PMC9214177

[20] Lambrou AS, Shirk P, Steele MK, Paul P, Paden CR, Cadwell B, et al.; Strain Surveillance and Emerging Variants Bioinformatic Working Group; Strain Surveillance and Emerging Variants NS3 Working Group. Genomic Surveillance for SARS-CoV-2 Variants: Predominance of the Delta (B.1.617.2) and Omicron (B.1.1.529) Variants - United States, June 2021-January 2022. MMWR Morb Mortal Wkly Rep. 2022;71:206–11. 10.15585/mmwr.mm7106a4 35143464 PMC8830620

[21] Shang W, Kang L, Cao G, Wang Y, Gao P, Liu J, et al. Percentage of asymptomatic infections among SARS-CoV-2 Omicron variant-positive individuals: a systematic review and meta-analysis. Vaccines (Basel). 2022;10:1049. 10.3390/vaccines10071049 35891214 PMC9321237

[22] Rogan WJ, Gladen B. Estimating prevalence from the results of a screening test. Am J Epidemiol. 1978;107:71–6. 10.1093/oxfordjournals.aje.a112510 623091

[23] Mizoue T, Yamamoto S, Konishi M, Oshiro Y, Inamura N, Nemoto T, et al. Sensitivity of anti-SARS-CoV-2 nucleocapsid protein antibody for breakthrough infections during the epidemic of the Omicron variants. J Infect. 2022;85:573–607. 10.1016/j.jinf.2022.08.015 35995310 PMC9391226

[24] Buss LF, Sabino EC. Intense SARS-CoV-2 transmission among affluent Manaus residents preceded the second wave of the epidemic in Brazil. Lancet Glob Health. 2021;9:e1475–6. 10.1016/S2214-109X(21)00396-X 34678180 PMC8525982

[25] Bloch EM, Kyeyune D, White JL, Ddungu H, Ashokkumar S, Habtehyimer F, et al. SARS-CoV-2 seroprevalence among blood donors in Uganda: 2019-2022. Transfusion. 2023;63:1354–65. 10.1111/trf.17449 37255467 PMC10525030

[26] He Z, Ren L, Yang J, Guo L, Feng L, Ma C, et al. Seroprevalence and humoral immune durability of anti-SARS-CoV-2 antibodies in Wuhan, China: a longitudinal, population-level, cross-sectional study. Lancet. 2021;397:1075–84. 10.1016/S0140-6736(21)00238-5 33743869 PMC7972311

[27] Murhekar MV, Bhatnagar T, Thangaraj JWV, Saravanakumar V, Santhosh Kumar M, Selvaraju S, et al.; ICMR serosurveillance group. Seroprevalence of IgG antibodies against SARS-CoV-2 among the general population and healthcare workers in India, June-July 2021: A population-based cross-sectional study. PLoS Med. 2021;18:e1003877. 10.1371/journal.pmed.1003877 34890407 PMC8726494

[28] Renaud C, Lewin A, Gregoire Y, Simard N, Vallières É, Paquette M, et al. SARS-CoV-2 immunoassays in a predominantly vaccinated population: Performances and qualitative agreements obtained with two analytical approaches and four immunoassays. Vox Sang. 2024;119:533–40; Epub ahead of print. 10.1111/vox.13625 38577957

[29] Macdonald PJ, Ruan Q, Grieshaber JL, Swift KM, Taylor RE, Prostko JC, et al. Affinity of anti-spike antibodies in SARS-CoV-2 patient plasma and its effect on COVID-19 antibody assays. EBioMedicine. 2022;75:103796. 10.1016/j.ebiom.2021.103796 34971970 PMC8714467

[30] Prete CA Jr, Buss LF, Buccheri R, Abrahim CMM, Salomon T, Crispim MAE, et al. Reinfection by the SARS-CoV-2 Gamma variant in blood donors in Manaus, Brazil. BMC Infect Dis. 2022;22:127. 10.1186/s12879-022-07094-y 35123418 PMC8817641

[31] Von Bartheld CS, Wang L. An explanation for reports of increased prevalence of olfactory dysfunction with Omicron: asymptomatic infections. J Infect Dis. 2023. 37697932 10.1093/infdis/jiad394 PMC11032248

[32] Whitaker BI, Walderhaug M, Hinkins S, Steele WR, Custer B, Kessler D, et al. Use of a rapid electronic survey methodology to estimate blood donors’ potential exposure to emerging infectious diseases: Application of a statistically representative sampling methodology to assess risk in US blood centers. Transfusion. 2020;60:1987–97. 10.1111/trf.15941 32743798 PMC7436713

