# Correction: Vol. 30, No. 8

**DOI:** 10.3201/eid3107.C23107

**Published:** 2025-07

**Keywords:** errata, erratum, correction

Figure 3 had several errors in Detection of Nucleocapsid Antibodies Associated with Primary SARS-CoV-2 Infection in Unvaccinated and Vaccinated Blood Donors (E. Grebe et al.). The article has been corrected online (https://wwwnc.cdc.gov/eid/article/30/8/24-0659_article).

