# Supplementary material for: Detection of Nucleocapsid Antibodies Associated with Primary SARS-CoV-2 Infection in Unvaccinated and Vaccinated Blood Donors
Source: Emerg Infect Dis. 2024 Aug;30(8):1621–30. doi: 10.3201/eid3008.240659 (PMC11286071; doi:10.3201/eid3008.240659)
Supplement: Appendix — Additional information about detection of nucleocapsid antibodies associated with primary SARS-CoV-2 infection in unvaccinated and vaccinated blood donors. [file 24-0659-Techapp-s1.pdf]

Article DOI: https://doi.org/10.3201/eid3007.240659

EID cannot ensure accessibility for supplementary materials supplied by authors. Readers who have difficulty accessing supplementary content should contact the authors for assistance.

Detection of Nucleocapsid Antibodies Associated with Primary SARS-CoV-2 Infection in Unvaccinated and Vaccinated Blood Donors

Appendix

Derivation of a Revised Non-Reactive versus Reactive Cutoff for the Ortho Anti-Nucleocapsid (anti-N) Total Ig Assay

To identify an optimal cutoff for serologic detection of infection using first the Roche anti-N total Ig assay and later the Ortho anti-N total Ig assay, we performed receiver operating characteristic (ROC) curve analyses using samples from blood donors classified as not previously infected with SARS-CoV-2 (controls, 'true negatives') and previously infected with SARS-CoV-2 (cases, 'true positives'). This analysis was performed based on samples collected cross-sectionally in the National Blood Donor Serosurvey (NBDS), without any clinical data or self-reported infection status, as described below.

To determine the optimal cutoff for the Roche anti-N total Ig assay, collected samples that had been tested in parallel with the Ortho anti-S and the Roche anti-N total Ig assays during monthly cross-sectional serosurveillance in the universal screening phase of the NBDS program (June to November 2020) were identified for optimization of the reactive/non-reactive cutoff of the Roche assay. Since samples were collected before widespread availability of vaccines, anti-S total Ig results were treated as independent indication of infection status (both positive and negative). The anti-S total Ig assay was treated as a 'gold standard' in this analysis, based on excellent performance demonstrated in a previous study (sensitivity of 95.8% and specificity of 100%), which was marginally better than the performance of the anti-N total Ig assay (1). Furthermore, the purpose of the analysis was to maximize sensitivity of the anti-N total Ig assay

in the context of VI, which had not been assessed in the prior analysis. A total of 25,065 cases and 30,110 controls were identified using anti-S total Ig results (Roche optimization sample set). The controls were supplemented with 432 samples collected during 2019 before the emergence of SARS-CoV-2. A ROC curve analysis was performed, with optimality defined as maximal Youden's J, i.e., an equal weighting of sensitivity and specificity. The optimal cutoff index (COI) on the Roche anti-N total Ig assay was COI≥0.205.

To determine the optimal cutoff for the Ortho anti-N total Ig assay, a similar sample set tested with the Ortho anti-N total Ig assay from before vaccine roll out was not available. NBDS samples collected after vaccines became available that had been tested with the Ortho anti-S total Ig assay and with both the Roche and Ortho anti-N total Ig assays were identified. For the 'Ortho optimization sample set' we identified 371 donors previously infected with SARS-CoV-2 (cases, i.e., 'true positives') based on reactivity on both anti-S total Ig (manufacturer's cutoff) and Roche anti-N total Ig (revised cutoff derived above to improve sensitivity). For this analysis, controls ('true negatives') were restricted to pre-pandemic samples – 1,248 results supplied by QuidelOrtho from in-house specificity testing performed in support of the assay's Emergency Use Authorization, supplemented with 200 pre-pandemic samples tested as part of a SARS-CoV-2 serology performance study (*1*). As in the analysis for the Roche assay, Youden's J was used to identify an optimal cutoff. The optimal cutoff on the Ortho anti-N total Ig assay was COI≥0.395.

Appendix Figure shows the distribution of S/CO values in the pre-pandemic control samples and the serologically defined cases (Ortho anti-S total Ig S/CO≥1 and Roche anti-N total Ig COI≥0.205), with vertical lines indicating the optimized cutoff based on our ROC analysis and the manufacturer's recommended cutoff.

References

1. Stone M, Grebe E, Sulaeman H, Di Germanio C, Dave H, Kelly K, et al. Evaluation of commercially available high-throughput SARS-CoV-2 serologic assays for serosurveillance and related applications. Emerg Infect Dis. 2022;28:672–83. PubMed https://doi.org/10.3201/eid2803.211885

2. Shang W, Kang L, Cao G, Wang Y, Gao P, Liu J, et al. Percentage of asymptomatic infections among SARS-CoV-2 Omicron variant-positive individuals: a systematic review and meta-analysis. Vaccines (Basel). 2022;10:1049. PubMed https://doi.org/10.3390/vaccines10071049

3. Jones JM, Manrique IM, Stone MS, Grebe E, Saa P, Germanio CD, et al. Estimates of SARS-CoV-2 seroprevalence and incidence of primary SARS-CoV-2 infections among blood donors, by COVID-19 vaccination status—United States, April 2021–September 2022. MMWR Morb Mortal Wkly Rep. 2023;72:601–5. PubMed https://doi.org/10.15585/mmwr.mm7222a3

Appendix Table 1. Sensitivity of Ortho anti-nucleocapsid (anti-N) assay for detection of first infections in vaccinated donors. Proportion reactive in the first sample collected after reported swab-confirmed infection, collected 14 to 180 d post-infection, stratified by whether an anti-S reactive/anti-N nonreactive sample was observed after vaccination but before infection

	First infections in vaccinated donors (S+/N- observed following vaccination)			First infections in vaccinated donors (No S+/N- following vaccination)		
Characteristic	No. donors	Manufacturer's cutoff,* % (95% CI)	Revised cutoff,† % (95% CI)	No. donors	Manufacturer's cutoff,* % (95% CI)	Revised cutoff,† % (95% CI)
Overall	5,079	95.5 (95.4–95.6)	97.1 (97.1–97.2)	3,108	95.8 (95.7–96.0)	96.8 (96.7–96.9)
Delta (Jul–Dec 2021)	103	77.7 (56.0–99.4)	82.5 (65.6–99.5)	1,246	95.3 (94.9–95.6)	96.3 (96.0–96.6)
Omicron (Jan–Dec 2022)	4,976	95.9 (95.8–96.0)	97.4 (97.4–97.5)	1,862	96.2 (96.0–96.4)	97.1 (96.9–97.3)
Age <65 y	3,004	96.3 (96.2–96.4)	97.6 (97.6–97.7)	2,190	96.0 (95.9–96.2)	96.8 (96.6–96.9)
Age ≥65 y	2,075	94.4 (94.1–94.7)	96.3 (96.2–96.5)	918	95.3 (94.8–95.8)	96.8 (96.5–97.2)
Symptomatic‡	4,691	96.0 (95.9–96.1)	97.6 (97.6–97.7)	2,725	96.5 (96.4–96.6)	97.4 (97.3–97.5)
Asymptomatic‡	322	89.4 (86.2–92.7)	90.4 (87.4–93.4)	305	90.8 (87.8–93.8)	92.1 (89.6–94.7)

*S/CO ≥1.000.
†S/CO ≥0.395.
‡Symptomatic or asymptomatic status could not be ascertained for all infections because of incomplete survey responses.

Appendix Table 2. Factors influencing anti-nucleocapsid seroconversion following first swab-confirmed SARS-CoV-2 infection. The table shows the proportion of first post-infection samples that were reactive using the standard cutoff on the Ortho assay, the unadjusted odds ratio, and the adjusted odds ratio obtained from multivariable logistic regression.

Variable	N	% detected	Unadjusted OR (95% CI)	Adjusted OR (95% CI)
Overall	14,094	93.9		
Variant era				
Delta	4,048	95.4	1.51 (1.28–1.78)	0.80 (0.66–0.98)
Omicron	10,046	93.3	*ref*	*ref*
Age Group (in years)				
16–29	554	96.0	2.13 (1.37–3.31)	1.83 (1.14–2.93)
30–49	3,582	95.3	1.80 (1.49–2.18)	1.41 (1.14–1.74)
50–64	5,671	94.3	1.44 (1.24–1.69)	1.23 (1.04–1.46)
65+	4,287	91.9	*ref*	*ref*
Gender				
Female	7,889	94.4	1.21 (1.05–1.38)	1.04 (0.89–1.20)
Male	6,205	93.3	*ref*	*ref*
Symptomatic infection				
Symptomatic	12,613	94.4	*ref*	*ref*
Asymptomatic	1,260	88.9	0.47 (0.39–0.57)	0.46 (0.37–0.57)
Vaccination status*				
Unvaccinated	3,927	96.6	*ref*	
Partially vaccinated	609	94.9	0.65 (0.43–0.97)	
Fully vaccinated	2,705	94.1	0.55 (0.43–0.70)	
Boosted	6,853	92.2	0.41 (0.34–0.50)	
Vaccination timing*				
≤1 mo	797	88.8	0.76 (0.58–1.00)	
2–3 mo	2,022	91.3	*ref*	
4–6 mo	2,617	92.7	1.21 (0.98–1.50)	
7–9 mo	2,652	94.5	1.64 (1.30–2.05)	
10+ months	2,079	94.0	1.50 (1.18–1.90)	
Vaccination status/timing (in days)				
Unvaccinated	3,927	96.6	*ref*	*ref*
Vaccinated (primary) ≤30	140	94.3	0.57 (0.28–1.20)	0.57 (0.26–1.24)
Vaccinated (primary) 31–180	999	94.4	0.59 (0.43–0.81)	0.63 (0.45–0.89)
Vaccinated (primary) >180	2,175	94.1	0.56 (0.43–0.71)	0.58 (0.44–0.75)
Vaccinated (boosted) ≤30	657	87.7	0.25 (0.19–0.33)	0.29 (0.21–0.40)
Vaccinated (boosted) 31–180	3,640	91.4	0.37 (0.30–0.46)	0.41 (0.32–0.52)
Vaccinated (boosted) >180	2,556	94.4	0.58 (0.46–0.74)	0.65 (0.50–0.85)
Time to sample				

Variable	N	% detected	Unadjusted OR (95% CI)	Adjusted OR (95% CI)
0–13 d	378	48.9	0.05 (0.04–0.06)	0.04 (0.03–0.05)
14–30 d	2,109	87.9	0.34 (0.29–0.40)	0.34 (0.28–0.40)
31–90 d	6,769	95.5	*ref*	*ref*
3–6 mo	3,539	97.5	1.85 (1.46–2.36)	1.79 (1.40–2.28)
7+ months	1,299	98.1	2.38 (1.58–3.59)	2.19 (1.44–3.35)

*Variables for vaccination status at time of infection, and for time from most recent vaccination to infection were replaced by a combined vaccination status and timing variable in multivariable logistic regression.

Appendix Table 3. Impact of adjustment for sensitivity and specificity, for detection of VI, on estimated percentage of vaccinated donors who became infected during three time periods in the National Blood Donor Cohort. We assumed that 32.4% of VI were asymptomatic (*2*) and we adjusted for symptom status-specific sensitivity estimates (see Methods).

Time period	No. infected / No. tested* among vaccinated, not previously infected	% infected % (95% CI) (naïve estimate)	% infected % (95% CI) adjusted for Se and Sp	% infected % (95% CI) adjusted for Se and Sp, assuming 32.4% of VI asymptomatic	Difference between adjusted and naïve estimate, % points (proportional difference), assuming 32.4% of VI asymptomatic
Q2 2021 to Q1 2022	7,656 / 33,815	22.64 (22.20–23.09)	23.18 (22.60–23.76)	23.52 (22.91–24.15)	0.88 (3.9%)
Q1 to Q2 2022	3,405 / 27,526	12.37 (11.99–12.76)	12.37 (11.80–12.91)	12.55 (11.96–13.11)	0.18 (1.5%)
Q2 to Q3 2022	5,569 / 24,147	23.06 (22.54–23.60)	23.63 (22.96–24.28)	23.98 (23.28–24.67)	0.92 (4.0%)

*The numerator is the number of donors who were classified as vaccinated and not previously infected in the previous quarter, who were tested and seroconverted anti-N by the following quarter, and the denominator is the number that seroconverted plus the number who did not seroconvert, without any demographic weighting (*3*).

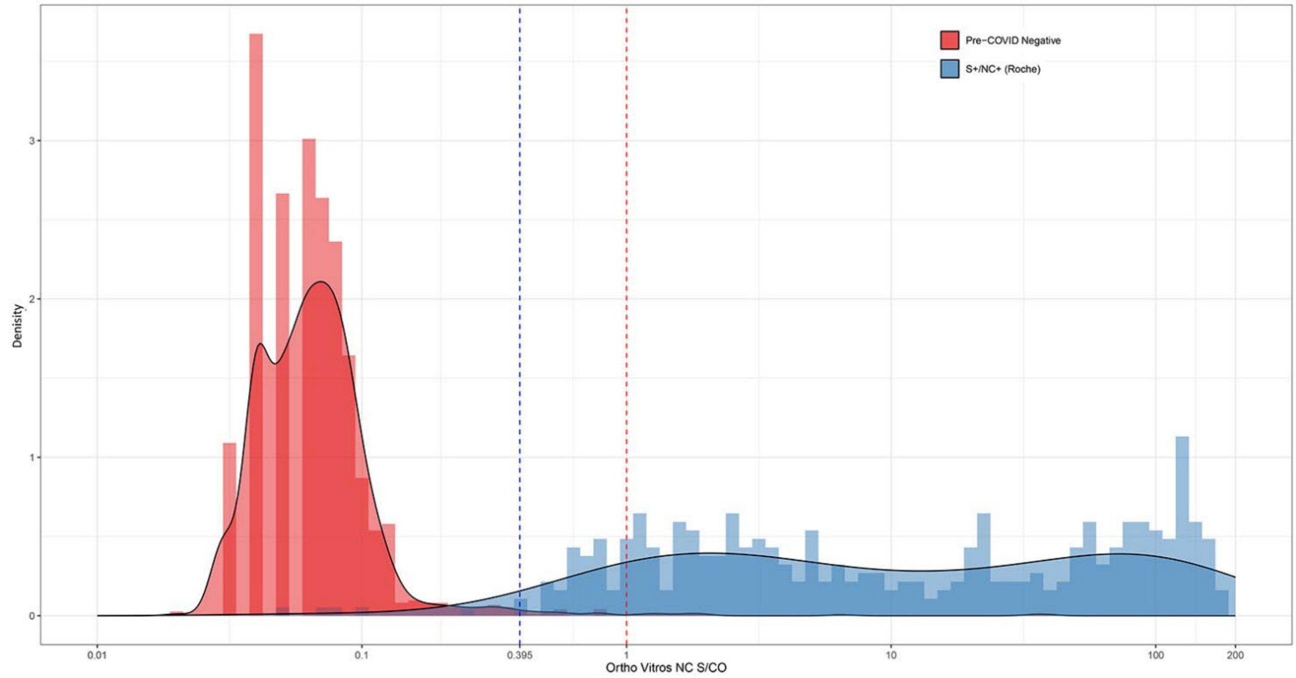

Appendix Figure. Anti-nucleocapsid (anti-N) signal-to-cutoff ratios on the Ortho anti-N total Ig assay, among pre-COVID-19 negative control samples, and samples from previously infected donors identified as anti-S and anti-N reactive using the Ortho anti-S and Roche anti-N total Ig assays. The red dashed line shows the manufacturer's recommended cutoff and the blue dashed line shows revised cutoff identified as optimal using receiver operating characteristic (ROC) curve analysis.
